# Interrelated Oncogenic Viruses and Breast Cancer

**DOI:** 10.3389/fmolb.2022.781111

**Published:** 2022-03-28

**Authors:** Samia Afzal, Khadija Fiaz, Afifa Noor, Amira Saleem Sindhu, Asma Hanif, Ayesha Bibi, Muhammad Asad, Saba Nawaz, Saba Zafar, Sidra Ayub, Syeda Bariyyah Hasnain, Muhammad Shahid

**Affiliations:** Center of Excellence in Molecular Biology (CEMB), University of the Punjab, Lahore, Pakistan

**Keywords:** breast cancer, human papilloma virus, Epstein–Barr virus, mouse mammary tumor virus, bovine leukemia virus

## Abstract

Breast Cancer is a multifactorial disease and recent evidence that viruses have a greater role in its aetiology and pathophysiology than previously hypothesized, has garnered a lot of attention in the past couple of years. After the role of Mouse Mammary Tumour Virus (MMTV) in the oncogenesis of breast cancer has been proved in mice, search for similar viruses found quite a plausible relation of Human Papilloma Virus (HPV), Epstein–Barr virus (EBV), and Bovine Leukaemia Virus (BLV) with breast cancer. However, despite practical efforts to provide some clarity in this issue, the evidence that viruses cause breast cancer still remains inconclusive. Therefore, this article aims to clarify some ambiguity and elucidate the correlation of breast cancer and those particular viruses which are found to bring about the development of tumorigenesis by a previous infection or by their own oncogenic ability to manipulate the molecular mechanisms and bypass the immune system of the human body. Although many studies have reported, both, the individual and co-existing presence of HPV, EBV, MMTV, and BLV in patient sample tissues, particularly in Western women, and proposed oncogenic mechanisms, majority of the collective survey of literature fails to provide a delineated and strong conclusive evidence that viruses do, in fact, cause breast cancer. Measures to prevent these viral infections may curb breast cancer cases, especially in the West. More studies are needed to provide a definite conclusion.

## Introduction

Breast cancer is a chronic disease, primarily owing to its metastatic characteristics, due to which it can relocate to other parts of the body such as bone, liver, brain and lungs. With 2.3 million women diagnosed, and 685,000 worldwide deaths in the year 2020 alone, breast cancer continues to retain its place as the most prevalent cancer in the world (Breast Cancer, 2021). The incidence of breast cancer in Pakistani women is increasing at an alarming rate of 19.33% every year, owing to the fact that one in every nine woman suffers from it (Baloch et al., 2012). Due to this, the ratio of breast cancer in Pakistani women is the most in the whole of Asia (Baloch et al., 2012).

Breast cancer is becoming increasingly common in women, as a 150 times higher number of cases are reported in women as compared to men (Lawson, 2009). However, it also occurs as a rare malignancy in adult older males, with approximately 1% reported cases (Siegel et al., 2016). The liability of occurrence and mortality of breast cancer is four to five-fold higher in Western women as compared to Eastern, particularly, Asian women (Ghocheh et al., 2015). Moreover, high mortality rates are observed in less developed countries as compared to more developed countries, which have a high prevalence (Ghocheh et al., 2015).

A large number of internal and external factors bring about the development of breast cancer. Some significant external factors that run the risk of inducing breast cancer include: genetic susceptibility (e.g., mutations in BRCA1/2 and other genes), obesity, a familial history of breast cancer, unhealthy behavioural choices, hormonal contraception and treatment after menopause (Lawson, 2009). Besides genetics, some other external factors that can contribute to breast cancer initiation are variation of food consumption among populations, and difference in fertility pattern (Lawson, 2009).

However, among such factors, despite that around 16% of all human cancers are caused by biological role carcinogens, the role of oncogenic viruses is less obvious (de Martel et al., 2012). Since a couple of decades, the evidence that viruses may play an influential role in the pathogenesis of breast cancer is becoming increasingly clear. Among such oncogenic viruses, the role of Human Papilloma Viruses (HPVs), Epstein–Barr virus (EBV-also known as human herpes virus type 4), Mouse Mammary Tumour Virus (MMTV) and Bovine Leukemia Virus (BLV) particularly stand out. In recent literature, reported information regarding the presence of viruses and their manipulation of molecular mechanisms for tumorigenesis is somewhat ambiguous and contradictory, and remains yet to be annotated (Lawson et al., 2018). Therefore, the aim of this article is to shed some light on the correlation of the presence of HPV, EBV, MMTV and BLV, their infections, and their oncogenic ability by which they exploit the human molecular pathways and the immune system to bring about the pathogenesis of breast cancer.

## Human Papilloma Virus and Breast Cancer

**FIGURE 1:**
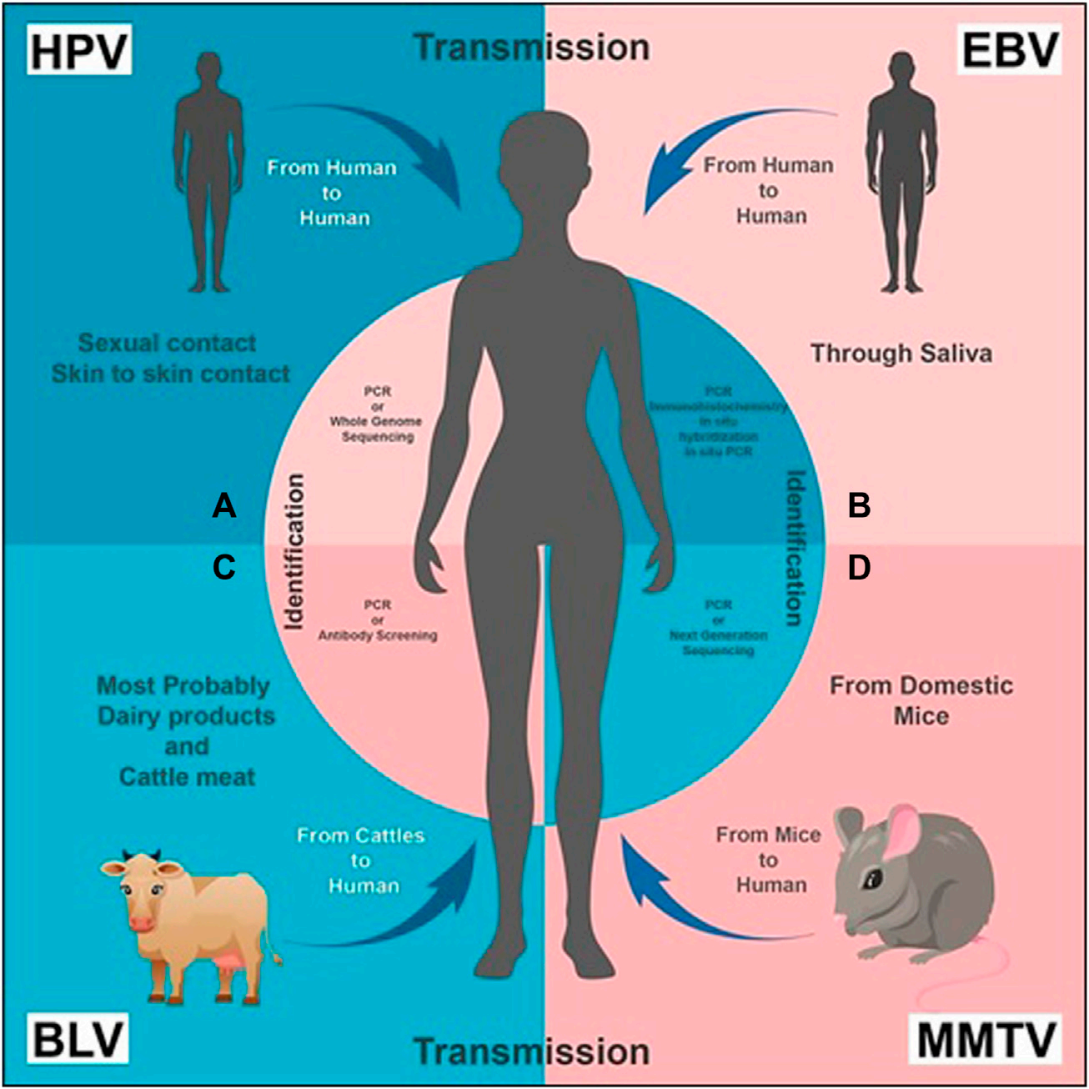
Transmission and Identification of the viruses, respectively: **(A)** HPV transmission takes place from human to human through sexual and/or skin to skin contact. It can be diagnosed by PCR (recommended) or Whole Genome Sequencing. **(B)** EBV is transmitted through saliva from human to human. Its presence can be identified by PCR, immunohistochemistry, *in situ* hybridization and *in situ* PCR. **(C)** BLV transmission primarily takes place from bovine or cattle (usually domestic animals) to humans; dairy and cattle meat can also be a source of transmission. PCR or antibody screening of a human blood sample is carried out for its diagnosis. **(D)** MMTV is transmitted from mice to human. Its presence is identified by PCR or NGS.

Human Papilloma Virus (HPV) is small in size, circular and contains double stranded DNA as its nucleic acid. About 200 different species of HPVs are classified into two categories: mucosal and cutaneous (Münger et al., 2004). Infection due to HPV is majorly transmitted through sexual activities or by skin-to-skin contact (Figure 1A). These are mostly short lived and, within 2 years of infection, 90% of men and women naturally clear the viral load (Winer et al., 2003).

There is evidence that high risk HPV 16 and 18 are involved in cervical carcinoma and other cancers related to genital sites (Shukla et al., 2009). Most of HPV strains are causative agents of cervical cancer, but “high risk” HPVs have been reported for breast cancer, as well (Rassi et al., 2009). HPV-associated cervical infections can precede the development of same type HPV-positive breast cancer in the same patient (Lawson et al., 2016) As a result, HPV viral load is higher in cervical cancer as compared to that in breast cancer (Khan et al., 2008). Due to this, identification of HPV in breast tumours is quite difficult, but it can be done either by whole-genome sequencing, or by PCR (Figure 1A). High-risk HPVs have also been identified in the SK-BR-3 breast cancer cell line (Yan et al., 2016). In a study, whole-genome sequencing was used to identify high-risk HPVs in invasive breast cancers (Lawson et al., 2015).

**FIGURE 2:**
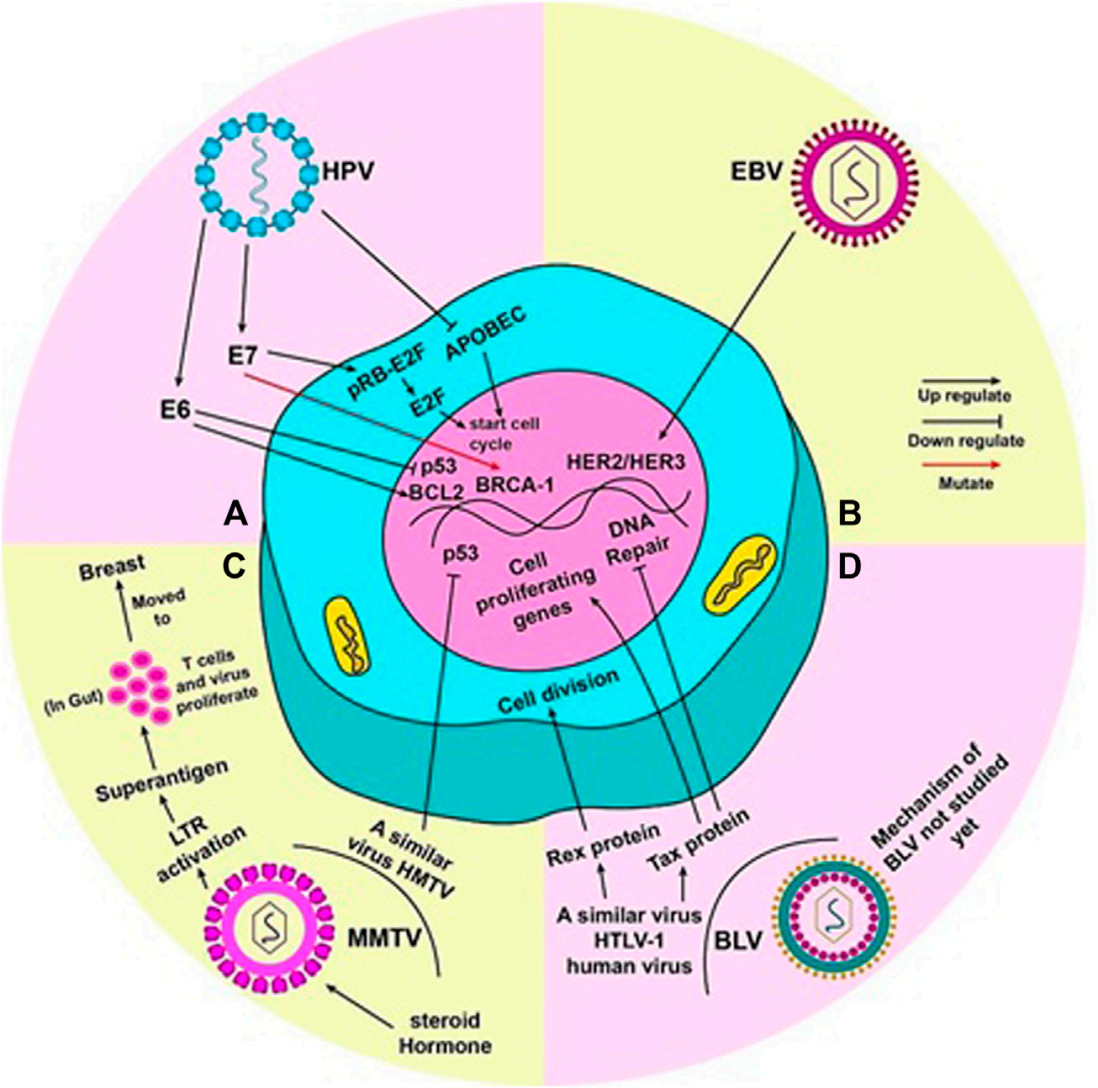
Mechanism by which each virus is said to cause breast cancer: **(A)** When mutated, oncoproteins (E6 and E7) of HPV virus activate pro-tumor genes. E6 activates BCL2 and inhibits p53, thus, promoting cell division. The pRB-E2f complex inhibits cell cycle; E7 detaches E2f from pRB and E2F alone commences the cell cycle. Mutation of BRCA-1 gene by E7 can also lead to tumor development. HPV also inhibits APOBEC (a cell cycle inhibitory molecule) to start cell cycle and cause genome instability. **(B)** EBV can activate HER2/HER3 gene(s) to render cell towards uncontrolled division. **(C)** Steroid hormones lead to efficient replication and increased expression of MMTV genome. An LTR in the MMTV genome codes for superantigens which activate T lymphocytes. MMTV replicates rapidly in these T cells in the gut, during which it can be carried to the breast. HMTV, a virus similar to MMTV, can inhibit p53 to induce to cell cycle. **(D)** The true mechanism by which BLV causes cancer in humans has not been yet studied. However, HTLV-1, a virus similar to BLV present in humans, can lead to uncontrolled cell division by the production of Rex and Tax proteins. Tax protein accentuates this by activating cell proliferating genes and halting DNA repair mechanism of the cell.

Duct epithelia are the rising ground in most breast cancers; similarly, HPV is reported to enter mammary ducts through the nipple areola complex, which is continuously exposed to the external environment (Polyak, 2007). The cell cycle control enzyme, APOBEC, is altered by viral infection, which causes genomic instability and leads to breast cancer (Ohba et al., 2014). Some studies also report that the early genes of oncoproteins E6 and E7 of HPV 16 inactivate the two major tumour suppressor proteins of the human body, Rb and p53, by interacting with them (Dyson et al., 1989; Werness et al., 1990). This is reported to commence continuous division of mammary epithelial cells in *in vitro* experiments, and in doing so, immortalize them, leading to breast cancer (Wazer et al., 1995; Liu et al., 1999). According to reports of familial breast cancer cases, these oncoproteins are notorious for inducing mutations in *BRCA1* gene, due to the functional interaction that exists between E7 and the *BRCA1* gene (Rassi et al., 2009). E7 of high risk HPV 16 dissociates the pRB-E2F complex by binding to pRB (Zhang et al., 2005). Lower p53 and higher BCL2 level is a hallmark of breast carcinogenic tumours. E6 deregulates expression of p53 and BCL2 by inactivating p53 and releasing repression of BCL2 (Figure 2A) (Sidransky and Hollstein, 1996).

In addition, a research study of the Indian population also observed the role of “high risk” HPV 16 in promoting breast cancer at genetic and epigenetic levels (Islam et al., 2017). They found that frequent integration of HPV into host’s genome is seen through disruption of hinge region (E28) of E2 gene, which gradually increases at different stages. Even lower viral loads are associated with cancer induction. Moreover, they confirmed HPV occurrence by sequencing and analysing LCR, E6 and E7 regions of HPV 16 genome. Variation analysis of LCR revealed one common variant (7521G>A) that overlaps with the binding of transcriptional repressor, YY1, regulating the expression of E6 and E7, and four novel variants (7628A>T, 7800A>G, 7837A>G and 7839A>C). The variant of E6 (350T>G) has been shown to be associated with HPV pathogenicity, while the two novel variants (519G>A) and (395G>T), destabilize the E6 mRNA and protein stability. The integration of HPV 16 in breast cancer is also linked to hyper methylation of promoter and enhancer regions of P97. Furthermore, expression analysis of E6 and E7 of HPV 16 showed two spliced transcripts (E61* and E6II*) of E6 and three spliced transcripts (E6*I/E7, E6*II/E7, E6^E7) of E6/E7, along with two novel fusion transcripts R6^E7*I and E6^E7*II in samples of breast cancer hypothesized to be associated with HPV (Islam et al., 2017).

In concordance with results of Ohba et al. (2014), another study suggested that HPV also influences genomic instability by interfering with the cell cycle control enzyme APOBEC, ultimately leading to breast cancer. They also observed that HPV initiates cancer and then disappears from tumour cells by the time cancer is clinically detected, therefore, suggesting a “hit and run” hypothesis for its initial mode of action (Balci et al., 2019).

Reports regarding the causal role of HPV and breast cancer seem to be contradictory in nature. Some studies report the presence of HPV in breast tumour samples either before or during the confirmation of the disease. For example, a study conducted in Iranian females with breast cancer discovered that 25.9% of tumour samples were positive for HPV DNA, and among these, 53.34% were of HPV 16 and 18 (Sigaroodi et al., 2012). Similarly, Salman et al. (2017) reported selective presence of twelve high risk HPVs in 42% of 110 female breast tumour samples, and confirmed viral activity majorly in invasive carcinomas. However, some studies report no or very low expression of HPV in samples associated with breast cancer. For instance, a study executed on Danish women with breast cancer, who were previously affected with cervical dysplasia, found only 1.55% prevalence of HPV in the assays they used (an in-house semi-Q PCR assay and SPF_10_ PCR-DEIA-LiPA_25_) and no particular difference regarding between samples of patients and controls (Bønløkke et al., 2018). Furthermore, from a samples size of 76 breast carcinomas and two benign tumour samples in a research study Spain, no evidence of HPV genome was found by Vernet-Tomas et al. (2015).

Although breast cancer cannot be prevented, one can and must observe some strategies to minimize its risk. Therefore, it's recommended for women to regularly perform self and screening exams, as well as getting frequent mammograms with advancing age (Purdie, 2019). The best way to prevent an HPV infection is to get vaccinated against it. FDA approved drugs for HPV are classified as: human papillomavirus bivalent vaccine (Cervarix); human papillomavirus Quadrivalent vaccine (Gardasil); human papillomavirus 9 violent vaccine (Gardasil 9) (Purdie, 2019). The shots of vaccination differ in Gardasil being specific for ages 9–14, while Gardasil 9 is for people aged 15–26 (Purdie, 2019). According to a survey study, Gardasil 9 protects against nine types of HPV infection, which hold prime importance against genital warts, while the other seven types are fruitful against other cancers caused by HPV, including breast cancer (HPV and Cancer National Cancer Institute, 2021).

Moreover, various other important aspects to treat HPV-related breast cancer are also present. Focusing on molecular targets which facilitate gene therapy is one such way; for example, a recent study showed that knockdown of HPV 18’s E6 and E7 oncogene results in inhibition of cancer progression (Yan et al., 2016). Similarly, finding alternative ways of breast cancer detection when target receptors are absent in patients is another useful strategy. For instance, a study reported 15% positivity of HPV in triple negative breast cancer (an aggressive form of breast with poor prognosis), which lacks receptors for ER and PR genes and shows an inability to amplify expression of *HER2* gene (Abramson et al., 2015; Horakova et al., 2018).

## Epstein–Barr Virus and Breast Cancer

Epstein-Barr virus (EBV), a gamma-Herpes virus which belongs to the Herpesviridae family, has a linear double-stranded DNA genome that encodes more than 85 genes. It is commonly found in approximately 95% of the total population. Approximately, 200,000 malignancies all over the world are annually associated with EBV. Its infection is common worldwide, but usually appears later among Western individuals in comparison to the developing communities (Speck and Longnecker, 2000). The most common route of transmission of the Epstein–Barr virus is saliva (Figure 1B). Primary infection of EBV causing a mild disease is more frequently reported in childhood. It appears asymptomatic in 20%–80% individuals aged two or three (Bolis et al., 2016; Prabhu and Wilson, 2016). When exposed to EBV, approximately 30%–70% of healthy young individuals can develop infectious mononucleosis (Bolis et al., 2016).

In addition to mononucleosis, EBV is reported as the causative agent of lymphoma, Langerhans cell histiocytosis, and nasopharyngeal cancer (Kang and Kieff, 2015). It is also responsible for oncogenesis in humans, and causes a latent infection that persists lifelong in most afflicted people (Mazouni et al., 2015). A restricted expression of viral proteins (latency III: six nuclear proteins EBNA-1, EBNA-2, EBNA-3A-C, EBNA-LP, and three latent membrane proteins (LMP-1, LMP-2A, and LMP-2B)) characterize its latent infection (Kang and Kieff, 2015). Along with lymphomas, EBV is associated with several other cancers, especially breast cancer, in which it is reported to induce tumour formation only by cell-to-cell contact (Speck and Longnecker, 2000). Techniques like immunohistochemistry, *in situ* hybridization, standard liquid PCR, and *in situ* PCR have been used to identify EBV genes in breast cancer in various countries (Figure 1B) (Hu et al., 2016). Similar methods found out the expression of EBV in some benign breast tissues, and its gene sequences have been identified even before the development of EBV-positive breast cancer. The prior and later specimens were from the same patients (Hu et al., 2016).

The most likely mechanism proposed by which EBV can form tumours is the one in which it activates the *HER2/HER3* signalling cascades, resulting in infection predisposition in breast epithelial cells, leading to the dreaded malignant transformation stage (Figure 2B). *HER2* and *HER3* are oncogenes that are known to be involved in the development of human breast cancer with a poor prognosis. CD21 receptors are also involved in transforming primary mammary epithelial cells to malignant cells by EBV infection and are no longer needed by the cells after onset of malignancy (Hu et al., 2016).

Due to the low reported frequency of BRCA1 and BRCA2 genes in Sudanese women with breast cancer, the methylation status of six tumour suppressor genes (*BRCA1*, *BRCA2*, *p16*, *p14*, *MGM2*, and *hMLH*) was observed by Liu et al. (2012). The reported methylation frequencies of the genes were: 84% of *BRCA1*, 84% of *BRCA2*, 15% of *p16*, 81% of *p14*, 12% of *MGM2*, and 18% of *hMLH*. A potent effect of the EBV virus on the methylation machinery is suggested by the high frequency of epigenetic silencing in *BRCA1*, *BRCA2*, and *p14*; this is an oncogenic mechanism delineated in other cancers except in breast cancer (Ryan et al., 2010; Liu et al., 2012). The methylation of *p14* can be a potential mechanism by which the tumour genome evades control of p53 and other related tumour suppressors, thereby, annulling the role of gene mutations. Esteller et al. (2000) first proposed this mechanism in colorectal cancer, and its basis was the over-representation of p14ARF hyper-methylation in tumours with wild-type p53, in comparison to tumours harbouring p53 mutations. Tumour type greatly influences the methylation profiles of tumour suppressor genes, and each tumour is designated with a distinct “DNA hypermethylome” (Estellar, 2005).

Lin et al. (2007) infected MCF7-A and BT474-A cells with EBV, and increased anchorage-independent growth was observed in cells on soft agar. This increase in cell mass was found to be associated with hyper-activation and expression of *HER2/HER3* signalling cascades, as supported by the findings that the treatment of phosphatidylinositol 3-kinase inhibitor, *HER2* antibody trastuzumab (Herceptin), or MEK inhibitor completely ceased the oncogenic capacity. The expression of EBV latency genes *EBER1*, *BARF0*, and *EBNA* was determined in breast cancer cells infected with EBV. However, it has been detected that *BARF0* alone was enough to promote tumorigenic activity in MCF7 and BT474 cells by up-regulating the expression of *HER2/HER3*, utilizing the techniques of small interfering RNA knockdown and overexpression. Consequently, a significant rise in the HER2 protein level was also reported. Yarden and Sliwkowski (2001) stated that HER2 lacks an activating ligand; therefore, it forms heterodimers with HER1, HER3, or HER4 to transfer signals downstream upon ligand binding. The expression status of HER1, HER3, and HER4 was also examined, and it was found that EBV-infected cells showed significantly increased expressions of HER3 at both transcription and translation levels, whereas, the expression of HER1 and HER4 was unaffected. A dramatic increase of tyrosine phosphorylation of HER2 coupled with increased binding of p85 (PI3K subunit) to HER2/HER3 complex was also reported. All these results suggest that EBV infection enhances both the expression and functional activity of HER2/HER3. Collectively, it was demonstrated that EBV-encoded *BARF0* promotes the oncogenic capacity of breast cancer cells by activating the HER2/HER3 signalling cascades.

Since the 1990’s, studies have reported the presence of EBV in human B lymphocytes, by which the virus has an easier access to breast epithelial cells for infection (Magrath and Bhatia, 1999). This might make it possible for the virus to commence oncogenesis in those breast epithelial cells (Richardson et al., 2015). However, contradictory results have been gained in regard to this hypothesis. For example, one of the first studies conducted in London centring on the presence of EBV in breast tumour samples showed a positive EBV result (Labrecque et al., 1995). Another study conducted in France reported the presence of EBV in 33.2% breast cancer tumours, which tended to be found in more aggressive cancer types (Mazouni et al., 2011). A very conflicting meta-analysis study reported that the highest prevalence of EBV-associated breast cancer was majorly present in Asia (35.25%), while the least was in the United States (18.27%) (Hou et al., 2012). Some studies report otherwise; Perrigoue et al., (2005) reported no contribution of EBV to breast cancer in their study conducted in America; Herrmann and Niedobitek (2002) found a negative association of EBV with breast cancer; in Mexico, Morales-Sanchez et al. (2013) took 86 tumour tissue samples and 65 tissues adjacent to the site of tumour and checked for the presence of EBV and MMTV to report that conventional PCR results showed no presence of either genome in any of the samples, while a more sensitive test with nested PCR revealed only four tumour samples to be positive for EBV.

Herpes viruses (a family of DNA viruses) predominantly express viral miRNAs (Cullen, 2009). Several viral mRNAs are targeted by viral miRNAs, for example, EBV encodes miR-BART2 that inhibits BALF5 (an EBV DNA polymerase) (Pfeffer et al., 2004). As described in several studies, the use of CRISPR/Cas9 technology interferes with the maintenance/replication of EBV, or edits its genome. CRISPR/Cas9 was used by Yuen et al. (2015) to delete a 558 bp sequence in the promoter region of the BamHIA rightward transcript via NHEJ, in virus-infected cells by direct chopping of the EBV genome. A transgene was introduced by HDR upon co-delivery of a template sequence at the same target site (Yuen et al., 2015). EBV-negative Akata cells can be successfully manipulated by the introduction of recombinant viruses (van Diemen et al., 2016). In addition to the application of CRISPR/Cas9 for the direct production of EBV variants, the use of anti-EBV gRNAs to hamper latent EBV infection has been described by two reports (Wang and Quake, 2014; van Diemen et al., 2016). Wang and Quake (2014) have used anti-EBV gRNAs to target the latent EBV genome in Raji Burkitt lymphoma cells by restricting viral replication and cell proliferation. gRNAs were constructed with the purpose to target various different regions including the viral genes *EBNA1*, *EBNA3C*, and *LMP1*of the latent EBV genome. The authors reported to achieve a reduction of 65%–85% in EBV viral DNA, as well as the arrest of cell proliferation. Moreover, complete removal of the latent virus has been shown in a subset of cells that express CRISPR/Cas9 (Wang and Quake, 2014; van Diemen et al., 2016). Single gRNAs successfully resulted in up to 50% loss of EBV genomes, whereas, two gRNAs resulted in up to 95% reduction in latent EBV-positive B cells (van Diemen et al., 2016). These two studies depict that with the CRISPR/Cas9 system, the dsDNA EBV episome can be successfully targeted and removed from latently infected cells. However, *in vivo* experiments in EBV infection models, for example, humanized mice are required to analyse the therapeutic potential of this approach.

## Mouse Mammary Tumour Virus and Breast Cancer

Mouse Mammary Tumour Virus (MMTV) is a perpetuating oncogenic virus which induces a set of familiar proto-oncogenes, causing the up-regulation of the promoter region and facilitating tumorigenesis via a protein production (Schrauzer, 2006). It triggers breast cancer in mouse and transmits it either exogenously through milk, or endogenously through the germ line (Schrauzer, 2006). MMTV is also transmitted in humans from the domestic mice *Mus domesticus*, illustrating the socioeconomic impact of breast cancer in humans with high prevalence rate in poor people who live in an unhygienic environment (Figure 1D) (Stewart et al., 2000). MMTV sequences have been identified from the long terminal repeats (LTRs) of MMTV’s genome by using PCR and NGS technologies, but with more valid outcomes from the former (Figure 1D) (Narty et al., 2017). By combining hybridization with DNA cloning techniques, Callahan et al. (2012) found sequences related to MMTV in human breast cancer cells. However, Szakacs and Moscinski (1991) identified the whole provirus of MMTV with a typical retroviral structure e.g. LTRs, *gags*, *pol*, *env* and RTRs in 13% of tested human breast cancers. In 2019, (Naccarato et al., 2019) identified the presence of more MMTVels in sporadic breast cancer cells than in hereditary breast cancer cells. A characteristic dUTPase in the *gag* gene has recently been discovered that is essential for viral replication and can be used for the confirmation of MMTV in human breast cancer using primer studies (Hizi and Herzig, 2015; Lehrer and Rheinstein, 2019).

MMTV has been noted to replicate efficiently in the alveolar epithelial cells of the mammary glands and an increased expression of MMTV has been observed during the lactation period due to the release of steroid hormones (Taneja et al., 2009). MMTV contains a long terminal repeat (LTR) of 1.3 kb size, which is responsible to encode a protein (super-antigens) which, in turn, activates T lymphocytes (Amarante et al., 2019). This protein triggers the replication of virus and amplification of T lymphocytes, which acts as a carrier to transfer the virus from the gut to the breast (Figure 2C) (Amarante et al., 2019). Moreover, the malignant transformation of human breast cancer epithelial cells is also done by the protein termed as p14 being expressed on envelope gene of MMTV (Katz et al., 2005). This p14 protein has shown its oncogenic capability when overexpressed (Feldman et al., 2012). Some other proteins like Rem, Sag, Naf or uncharacterized analogs of Tax also play a vital role in the transformation of human BC epithelial cells (Lawson et al., 2018). A virus similar to MMTV, named as the human mammary tumor virus (HMTV), which is found to be a potential cause of human breast cancer, contains DNA sequences with a 95%–99% homology with MMTV enveloped gene; it is found in samples including BC like serum, saliva, milk and others (Amarante et al., 2019). There is a consistently low level of HMTV identification in normal and benign breast cancers than human breast cancers (Lawson and Glenn, 2019). HMTV DNA sequences are linked with p53 nuclear aggregation and the presence of receptors for progesterone, which causes the cellular down-regulation, leading to breast cancer (Schrauzer, 2006).

Samples of human breast tumors have been found to include MMTV sequences from the long terminal repeat (LTR) region of the MMTV genome. These MMTV *env* gene sequences are unique to the MMTV genome and are not found in human endogenous retrovirus (HERV) sequences, which are frequently found in human genome research. In any of the MMTV-like *env* positive DNAs, no MoMt or IAP DNA sequences were found. Wnt-1 expression is much higher in MMTV-like positive breast cancer specimens than in MMTV-like negative breast cancer specimens, which is consistent with studies showing high Wnt-1 expression in MMTV positive mouse mammary tumors. PyMT, a membrane scaffold protein, activates MAPK and PI3K pathways, which are involved in cell proliferation and survival, by boosting various signaling pathways, including Shc and PI3-kinase (Cai et al., 2017; Nartey et al., 2017).

Many of the strongly enriched pathways, such as ECM-receptor interaction and focal adhesion, are essentially portion of the large “pathway in cancer” (KEGG: 05200). The PyMT oncogenesis causal route PI3K-AKT signaling is also included in the “pathway of cancer,” and is connected to several of those enriched pathways. Deregulation of metabolism has long been associated with cancer, and a high-calorie diet has been shown to increase cancer risk, particularly in terms of glucose metabolism. Many metabolic related words and pathways, notably the TCA cycle pathway, were found to be overrepresented in both DEGs and WGCNA module genes (Cai et al., 2017; Nartey et al., 2017).

Although many studies have confirmed the presence of MMTV-associated breast cancer in mice, and have proposed a pathway by which MMTV can cause tumorigenesis in humans, some ambiguity is still present in this regard. Studies have reported both, the presence and absence of MMTV genome or its sequences in breast cancer samples. For example, studies of breast cancer in females only by using supplementation of Wang et al. (1995) has shown 15- fold more prevalence of MMTV-*env* sequences in human breast cancers than the control ones. Lawson and Glenn (2019) also detected 15-fold higher sequences similar to MMTV in samples of breast tumour than control samples and reported them to be 40% more in patient samples than normal controls. Serum of women with breast cancer was also found to contain a 5-fold higher presence of MMTV antibodies than normal women serum (Lawson and Glenn, 2019). They also located the same MMTV-like sequences in lactating women and in benign breast tumour tissues 1–11 years before diagnosis (Lawson and Glenn, 2019). They reported these MMTV-like sequences to be highly similar to MMTV (Lawson and Glenn, 2017). Likewise, a study executed in Saudi Arabia also detected the presence of MMTV-*env* provirus sequences in human breast tumour samples (Al Dossary et al., 2018). However, due to heterogeneous results, it is not a definitive conclusion. There is vast difference in prevalence of MMTV- like breast cancer according to the geographical distinction. It has been observed that breast cancer prevalence is much more in western countries (30–40%) than Asian countries (10%–20%) (Stewart et al., 2000). Approximately 16% variations in Mexico’s identification of MMTV breast cancers and 12% in Iraq has been observed and similar variation has been determined in United States, Italian and Australian breast cancers (Lawson et al., 2018). Furthermore, Hameed et al. (2020) declared that no association of MMTV and breast cancer was found in their study, in which they applied the Bradford Hill criteria and perused studies with both normal and benign samples along with those of breast cancer. They, however, reported that it could worsen the state of breast cancer after its tumorigenesis had already begun (Hameed et al., 2020). One more study conducted in Myanmar looked for the presence of Human Mammary Tumour Virus in breast cancer patients, which has a 90%–95% homology to MMTV and is mostly found in humans; from 58 patient samples, they found only one sample containing the HMV sequence, whose prevalence of 1.7% is obviously quite low (San et al., 2017).

Various drugs and chemicals have shown promising results in treatment of breast cancer and MMTV infection. For instance, Cathepsin D (CTSD) is a lysosomal protease marker that has shown fewer prognoses in human breast cancer by blocking tumor development in cell- independent way. Its absence delays the growth of tumor in human for 2 months. Proliferation of quiescent CTSD^−/−^ tumor cells has restarted upon long term culture by reiterating oncogenic gene expression and signaling pathways (Ketterer et al., 2020). Moreover, in an ecological and correlational study, it was revealed that selenium acts as an anti-tumorigenic agent when given to MMTV positive mice as a dietary element (Schrauzer, 2006). The traces of selenium present in the samples were shown to inhibit the infection caused by MMTV in mice, whereas, chromium was found as an element antagonistic to selenium (Schrauzer, 2006). When chromium is present in +3 oxidation state, it acts as a nutritive factor, while chromium in +6 oxidation state acts as anti-selenium agent, decreasing the inhibitory effect of selenium (Schrauzer, 2006). So, chromium and selenium were showed to have interactive effects on the growth and development of mouse mammary tumor virus in MMTV infected female mice (Schrauzer, 2006). The relevance of interaction between selenium and chromium in humans is still in debate, but analysis of blood samples of workers working in a polish tannery, living in a dusty environment, and getting exposure to chromium showed decreased level of selenium (Schrauzer, 2006).

## Bovine Leukaemia Virus and Breast Cancer

Bovine Leukaemia Virus (BLV) is responsible for causing bovine leucosis in cattle worldwide. It belongs to the family of Retroviridae and is closely related to human T-lymphotropic virus type-1 (HTLV-1) (Gao et al., 2020). The size of BLV ranges between 60 and 125 nm, having an enveloped capsid and a diploid positive sense RNA in its structure (Martinez Cuesta et al., 2018). In 1969, Janice Miller and co-workers first reported BLV in cattle (Buehring and Sans, 2020). It infects species close to cattle such as sheep, buffalo, and alpaca; however, the RNA genome of BLV has also been reported positive in human breast tissues (Buehring et al., 2019).

Recently a surge in female death has been observed due to breast cancer, so finding its etiological factors is necessary. In recent advanced studies, it is concluded that viruses are also a predisposing factor to cause cancer. Majorly MMTV, HPV, and EBV have been found responsible for this guilt (Lawson et al., 2018). In a few studies, BLV has also been considered a cause of breast cancer in females (Sinha, 2016). Although BLV is a zoonotic virus (found especially in cattle), some shreds of evidence show the presence of BLV in humans, too. Its presence has been detected and identified in breast cancer samples by RT-PCR, *in situ* PCR assay, ELISA, immunohistochemistry, *in situ* hybridization, and DNA sequencing (Figure 1C) (Khatami et al., 2020; Le et al., 2020). Most likely, the virus’s presence in women’s breast tissue and blood indicates its transmission from cattle to humans (Khalilan et al., 2019). Moreover, antibodies against BLV have been isolated in humans, which contribute to evidence of its transmission (Buehring et al., 2003).

The exact mechanism through which the transmission of BLV in humans takes place is unknown. However, relating some conditions can probably resolve this problem. There are several hypotheses which justify the presence of BLV in humans. According to some studies, consuming contaminated dairy products and meat of cattle positive with bovine leukemia virus could be a possible way of transmission in humans (Olaya-Galán et al., 2017). Vertical transmission has been found in cattle, which is evidence that the virus is present in their milk (Buehring et al., 2015). According to some researchers, consuming unpasteurized and undercooked meat in some areas of the world could cause transmission in humans (Figure 1C). Some hypothesize that before pasteurizing technique, this virus became part of the human species (Khatami et al., 2020).

Oncogene presence in a virus or activation of proto-oncogenes of cells is an essential step in converting normal cells into malignant cells. BLV neither has an oncogene, nor can it incorporate its gene into the cellular genome to activate proto-oncogene (Aida et al., 2013). Moreover, the presence of a receptor is significant for a viral infection to take place. In recent studies, the presence of a receptor for BLV on human cells is still unknown. It indicates the resistance of humans against BLV; however, a close relation of BLV with the human T-cell leukemia virus (HTLV-1) might justify its relatedness to breast cancer (Ban et al., 1999). HTLV-1 is a human origin virus causing defects in T cells lineage. Both of these viruses have a nearly similar mode of action to cause disease.

Malignancies of the breast are not caused by directly interacting with the host genome. Instead, BLV is supposed to cause defects in the mechanisms responsible for the repair of base-pair or other kinds of mutations, leading to oxidative damage. The unrecovered mutations eventually accumulate and lead to various cancers, such as that of lungs or breast (Khatami et al., 2020).The genome of BLV is characterized by the presence of essential genes (*pro*, *pol*, *env*, *gag*), accessory genes (*G4*, *R3*) and regulatory genes (*rex* and *tax*) (Martinez Cuesta et al., 2018; Bai et al., 2020). *Tax* is also an oncogene, and is translated into proteins called Tax. This protein interferes with a number of normal functions, and renders them anomalous. The tax encoding genes are responsible for aberration in the DNA excision repair mechanism, prevention of apoptosis, and the down regulation in the function of tumour suppressors. The presence of *tax* gene, along with the *rex* gene, is marked in the Px region, which is between the envelope gene and one of the long terminal repeats (LTRs), and is a similar region present in the genome of both of these viruses (Gao et al., 2020). The presence of tax protein is also observed evidently in HTLV-1.

Tax protein can supposedly convert normal cells into malignant cells, primarily by converting lymphocytes into immortal cells. Moreover, by acting as an activating agent for cell proliferating genes, and inhibiting the repairing process of DNA damage, Tax protein can enhance uncontrolled cell division (Figure 2D). Studies show a new mutant form of Tax protein has been identified that is named TaxD247G. This mutant form of Tax protein can enhance genes’ activation more than wild type (Aida et al., 2013). On the evidence of these functions of Tax protein, we can hypothesize that BLV can cause breast cancer in humans. Further studies are required to thoroughly understand the role of BLV in inducing breast cancer (Buehring et al., 2015).

Bovine Leukaemia Virus primarily causes infection in bovine animals or cattle. Despite this, there are studies which report the initiation of lymphosarcoma in 69 sheep ensuing contact with materials contaminated with bovine lymphosarcoma (Olsen and Baumgartener, 1976). This gives evidence that it can be responsible to commence oncogenesis in an organism other than bovine or cattle. There are also reports of it playing a causal role in tumour in other animals like rats, water buffaloes, goats, rabbits, etc. (Schwartz and Levi, 1994). Along these lines, many cases have been reported around the globe in which the presence of BLV has been confirmed in breast cancer patients. In the United States, BLV was observed in breast cancer tissues in 2015. In Brazil, using molecular and immunological techniques, BLV presence was also confirmed. A similar study conducted in Argentina indicates that BLV could also a possible cause of breast cancer. In India, a rise in milk consumption could be a likely cause of breast cancer (Gao et al., 2020). Moreover, in Australia and Colombia, BLV presence has been found (Giovanna et al., 2013; Buehring et al., 2017). In China and Japan, BLV has not been found in breast cancer samples (Zhang et al., 2016; Saito et al., 2020). The one reason which can justify the absence of virus in Chinese people is their lactose intolerance, due to which milk consumers are significantly low in China (Gao et al., 2020).

## Synergistic Oncogenic Relationship of Mouse Mammary Tumour Virus, Human Papilloma Virus and Epstein–Barr Virus in Breast Cancer

Many research studies show that multiple viruses co-exist in samples extracted from women afflicted with breast cancer. Glenn et al. (2012) found genome sequences of more than one virus (namely, from EBV, high-risk HPV and MMTV) co-habiting in about 72% of samples taken from women with breast cancer. Moreover, they also found that DNA extracted from 50 samples of women with invasive breast cancer contained EBV genome sequence in 68% of the samples, MMTV genome sequence in 78% of the samples, and high-risk HPV genome sequence in 50% of the samples (Glenn et al., 2012). Their results also showed that the common presence of both HPV and EBV was 38% (three- to four-fold higher) in 50 patients with breast cancer as compared to 10% of 40 normal control breast tissue samples (Glenn et al., 2012). Results of the study conducted by Naushad et al. (2017) were in concordance with the aforementioned results; PCR showed the prevalence of hish-risk HPV, EBV and MMTV genome sequences in the same samples of Pakistani women with breast cancer.

According to the studies conducted by Gupta et al. (2020) and Al Moustafa et al. (2016) co-infection of both, high-risk HPV and EBV, was found in 47% of Qatari and 32% of Syrian female breast cancer patients, respectively. Based on their own previous study conducted in 2015, Moustafa (2015) postulated that since E5 and E6/7 oncoproteins of HPV could initiate or enhance the progress of human oral carcinomas by a co-operative interaction with the LMP1 and EBNA1 oncoproteins of EBV, via the EMT event, a similar mechanism could follow the pathogenesis and metastasis of breast cancer. Another study conducted in cervical smears found that chances of HPV genome integration in host cells increased five- to seven-fold when EBV presence was found along with it, suggesting that genome instability caused by HPV was enhanced in its presence (Polz-Dacewicz et al., 2016). Moreover, it was also suggested that by producing a homolog of interleukin 10, known as the BCRF1 gene product, EBV might hinder one’s immune response to cells transformed by HPV (Szostek et al., 2009). It is possible that breast cancer pathogenesis might be enhanced by any of the mechanisms suggested.

## Discussion

There is some evidence that Human Papilloma Virus can lead to breast cancer, but it is insubstantial. Many studies have reported both the presence and absence of HPV in breast cancer samples. For example, a study found a 26% prevalence of HPV DNA in Argentinian women with breast cancer (Suarez et al., 2013). High risk HPVs, namely HPV 16, 18 and 31, were also detected in 16 of 72 Egyptian females afflicted with breast cancer (El-Sheikh et al., 2021). However, a study conducted in Indian women with breast cancer found no traces of either HPV DNA or E6 and E7 proteins, both, by conventional and real time PCR (Hedau et al., 2011). Similarly, another study done on patients with breast cancer in Iran found DNA of HPV in only eight out of the 98 patient samples, which is an extremely low prevalence; among HPV positive samples, 62.5% of DNA was of HPV 16 and 18 (Malekpour Afshar et al., 2018). In addition, Kwong et al. (2013) found no traces of HPV by PCR in their study of 102 breast cancer patient samples in Hong Kong.

Most of the accounts aforementioned reporting the presence of HPV genome in breast tumour samples is in Western females. There is also a higher viral load in patients of breast cancer as compared to controls. On the contrary, absence of HPV DNA or extremely low prevalence is mostly seen in Eastern particularly, Asian, females. Therefore, to get a better picture of the causal link between HPV and breast cancer, more studies should be conducted on Eastern women with breast cancer, especially those who were previously affected by HPV infections in their life.

Similar results have been reported in regard to EBV. A study conducted in France discovered that 51% of tumour samples contained the EBV genome, while 90% of healthy tissue samples taken from near the tumour site showed its absence (Bonnet et al., 1999). This indicates that EBV genome is significant in these oncogenic epithelial cells, as neighbouring normal cells showed no trace of it. Moreover, Zekri et al. (2012) detected the presence of EBV in 45% of Egyptian women with breast cancer and 28% of Iraqi breast cancer patients and also reported a more plausible relationship of EBV with increased aggressiveness of the cancer. Meanwhile, Dowran et al. (2019) found no traces of EBV in Iranian breast cancer patients. Likewise, Kadivar et al. (2011) found an absence of EBV in 100 breast cancer patient samples.

Most of the studies which support the hypothesis that breast can also be EBV associated are conducted in the West. On the other hand, reports which deny its association with breast cancer and present mostly in Asia. However, no conclusive statement can be given in this regard, as any EBV-associated breast cancer samples were not found in America and New Zealand, too. It is possible, though, that breast cancer can be worsened or become aggressive by the infection of EBV after tumorigenesis. In any case, more studies need to be executed to eliminate this conflicting result.

In concordance with HPV and EBV, there is substantial evidence that MMTV can cause cancer in human breast tissues, but the underlying mechanism which execute this are not yet clear and defined. The evidence reports includes a study carried out in Egypt aimed to detect the presence of *env* sequences in MMTV-like DNA in samples of familial and non-familial breast cancer; the prevalence they found was 70% in familial and 76% in non-familial women suffering from breast cancer (Loutfy et al., 2021). Furthermore, one more study reported 65.72% prevalence of MMTV sequences on 105 breast cancer patients and also suggested that be used as a biomarker for cancer invasion (Khalid et al., 2021). However, a study reported a contrasting result; they detected the presence of MMTV in human breast cancer samples, but due to inconsistency and a later confirmation of it being murine DNA, they concluded that no association was present between MMTV and human breast cancer (Perzova et al., 2017). Likewise, another study which took place in Iran reported that they found no traces of two sequences of MMTV-like DNA by nested PCR in any of their 300 breast cancer patient samples (Motamedifar et al., 2012).

As compared to HPV and EBV, more studies reported the presence of MMTV or MMTV-like sequences in breast cancer in Western countries as compared to Eastern countries. Moreover, since some studies enumerate that an increased frequency of MMTV-like sequences were found in patient samples as compared to control, suggests that it may enhance tumour induction. However, this gap in these findings certainly needs to be abridged to get a picture with more clarity. A concrete statement cannot be given with such contrasting studies also existing, and being continuously reported, around the world. Therefore, it is more prudent to report that MMTV or MMTV-like sequences may play causal role for breast cancer, but evidence regarding this is still inconclusive.

BLV-associated infection and cancer is usually found in bovine animals and cattle, but varying results regarding it are also seen in human breast cancer samples. For instance, a case control study carried out in Texas, America, reported a significant presence of BLV genome in tissues of women with breast cancer as compared to those women with benign tumour or were normal (Baltzell et al., 2018). To know if there was any associative relationship of BLV and HPV in causing malignant breast cancer, they also executed PCR and DNA hybridization tests; however, they found no traces of HPV in any of the samples (Baltzell et al., 2018). Similarly, Khatami et al. (2020) reported by their meta-analysis study that nine case control studies centring on the causal role of BLV for breast cancer confirm the notion. As aforementioned, some reports also propose contrasting results. This could be due to low milk consumption in the Chinese and Japanese population, as compared to other countries where there was a prominent association between BLV and breast cancer, as already mentioned above.

Collective survey of literature does suggest that an association between BLV and breast cancer is highly likely, but the evidence is still insufficient. Efforts to eradicate bovines, cattle and other animals from BLV might be one way to control its supposed effect on breast cancer.

## Conclusion

In our opinion, even though there is evidence that Human Papilloma Virus can cause cervical and oral cancers and is considered as a high risk factor for breast cancer, it is not sufficient to conclude that it alone is a causal factor for breast cancer. Moreover, evidence that Mouse Mammary Tumour Virus can lead to breast cancer is suggestive and comprehensive, but is not conclusive because its prevalence is higher in Western females as compared to women residing in the East. In addition, although a pathway leading to the pathogenesis of breast has been proposed for Epstein – Barr Virus, it is inadequate to conclude that it is one of the root factors for breast cancer. Similarly, even though there are suggested pathways by which Bovine Leukemia Virus can confer breast cancer and is suggested to be highly likely, it is insufficient to report it as conclusive, due to different reports in varying geographical distributions. However, evidence that presence of multiple viruses in specimens of breast cancer, is more detailed and suggestive. More studies are required to conclude that these viruses definitely lead to the malignancy that is breast cancer. Moreover, even if there is a definite causal relationship between viruses and breast cancer, control of these viral infections in the first place might curb reported cases.

## References

[B1] Abramson V. G. Lehmann B. D. Ballinger T. J. Pietenpol J. A. (2015). Subtyping of Triple-Negative Breast Cancer: Implications for Therapy. Cancer 121 (1), 8–16. 10.1002/cncr.28914 25043972 PMC4270831

[B2] Aida Y. Murakami H. Takahashi M. Takeshima S.-N. (2013). Mechanisms of Pathogenesis Induced by Bovine Leukemia Virus as a Model for Human T-Cell Leukemia Virus. Front. Microbiol. 4, 328. 10.3389/fmicb.2013.00328 24265629 PMC3820957

[B3] Al Dossary R. Alkharsah K. R. Kussaibi H. (2018). Prevalence of Mouse Mammary Tumor Virus (MMTV)-like Sequences in Human Breast Cancer Tissues and Adjacent normal Breast Tissues in Saudi Arabia. BMC Cancer 18, 170. 10.1186/s12885-018-4074-6 29426297 PMC5810194

[B4] Al Moustafa A.-E. Al-Antary N. Aboulkassim T. Akil N. Batist G. Yasmeen A. (2016). Co-Prevalence of Epstein-Barr Virus and High-Risk Human Papillomaviruses in Syrian Women with Breast Cancer. Hum. Vaccin. Immunother. 12 (7), 1–4. 10.1080/21645515.2016.1139255 27082145 PMC4964818

[B5] Amarante M. K. de Sousa Pereira N. Vitiello G. A. F. Watanabe M. A. E. (2019). Involvement of a Mouse Mammary Tumor Virus (MMTV) Homologue in Human Breast Cancer: Evidence for, against and Possible Causes of Controversies. Microb. Pathog. 130, 283–294. 10.1016/j.micpath.2019.03.021 30905715

[B6] Bai L. Hirose T. Assi W. Wada S. Takeshima S.-n. Aida Y. (2020). Bovine Leukemia Virus Infection Affects Host Gene Expression Associated with DNA Mismatch Repair. Pathogens 9 (11), 909. 10.3390/pathogens9110909 PMC7694100 33143351

[B7] Balci F. L. Uras C. Feldman S. M. (2019). Is Human Papillomavirus Associated with Breast Cancer or Papilloma Presenting with Pathologic Nipple Discharge? Cancer Treat. Res. Commun. 19, 100122. 10.1016/j.ctarc.2019.100122 30785026

[B8] Baloch A. H. Shuja J. Daud S. Ahmed M. Ahmad A. Tareen M. (2012). Various Aspects, Patterns and Risk Factors in Breast Cancer Patients of Balochistan. Asian Pac. J. Cancer Prev. 13 (8), 4013–4016. 10.7314/apjcp.2012.13.8.4013 23098509

[B9] Baltzell K. A. Shen H. M. Krishnamurthy S. Sison J. D. Nuovo G. J. Buehring G. C. (2018). Bovine Leukemia Virus Linked to Breast Cancer but Not Coinfection with Human Papillomavirus: Case-Control Study of Women in Texas. Cancer 124 (7), 1342–1349. 10.1002/cncr.31169 29266207

[B10] Ban J. Hlavaty J. Orlik O. Splitter G. A. Altaner C. (1999). The Human Homologue of the Bovine Leukemia Virus Receptor BLVRcp1 is the δ-subunit of Adaptor-Related AP-3 Protein that Does Not Bind the BVLgp51. Arch. Virol. 144 (10), 2013–2022. 10.1007/s007050050722 10550673

[B11] Bolis V. Karadedos C. Chiotis I. Chaliasos N. Tsabouri S. (2016). Atypical Manifestations of Epstein-Barr Virus in Children: A Diagnostic challenge. J. Pediatr. 92 (2), 113–121. 10.1016/j.jped.2015.06.007 26802473

[B12] Bønløkke S. Blaakær J. Steiniche T. Høgdall E. Jensen S. G. Hammer A. (2018). Evidence of No Association Between Human Papillomavirus and Breast Cancer. Front. Oncol. 8, 209. 10.3389/fonc.2018.00209 29938198 PMC6002490

[B13] Bonnet M. Guinebretiere J.-M. Kremmer E. Grunewald V. Benhamou E. Contesso G. (1999). Detection of Epstein-Barr Virus in Invasive Breast Cancers. J. Natl. Cancer Inst. 91 (16), 1376–1381. 10.1093/jnci/91.16.1376 10451442

[B14] Breast Cancer (2021). World Health Organization. Available at: https://www.who.int/news-room/fact-sheets/detail/breast-cancer/ (Accessed on May 2, 2021).

[B15] Buehring G. C. DeLaney A. Shen H. Chu D. L. Razavian N. Schwartz D. A. (2019). Bovine Leukemia Virus Discovered in Human Blood. BMC Infect. Dis. 19 (1), 297. 10.1186/s12879-019-3891-9 30940091 PMC6444872

[B16] Buehring G. C. Philpott S. M. Choi K. Y. (2003). Humans Have Antibodies Reactive with Bovine Leukemia Virus. AIDS Res. Hum. Retroviruses 19 (12), 1105–1113. 10.1089/088922203771881202 14709247

[B17] Buehring G. C. Sans H. M. (2020). Breast Cancer Gone Viral? Review of Possible Role of Bovine Leukemia Virus in Breast Cancer, and Related Opportunities for Cancer Prevention. Int. J. Environ. Res. Public Health 17 (1), 209. 10.3390/ijerph17010209 PMC6982050 31892207

[B18] Buehring G. C. Shen H. M. Jensen H. M. Jin D. L. Hudes M. Block G. (2015). Exposure to Bovine Leukemia Virus Is Associated with Breast Cancer: a Case-Control Study. PLoS One 10 (9), e0134304. 10.1371/journal.pone.0134304 26332838 PMC4557937

[B19] Buehring G. C. Shen H. Schwartz D. A. Lawson J. S. (2017). Bovine Leukemia Virus Linked to Breast Cancer in Australian Women and Identified before Breast Cancer Development. PLoS One 12 (6), e0179367. 10.1371/journal.pone.0179367 28640828 PMC5480893

[B20] Cai Y. Nogales-Cadenas R. Zhang Q. Lin J.-R. Zhang W. O’Brien K. (2017). Transcriptomic Dynamics of Breast Cancer Progression in the MMTV-PyMT Mouse Model. BMC Genomics 18 (1), 185. 10.1186/s12864-017-3563-3 28212608 PMC5316186

[B21] Callahan R. Mudunuri U. Bargo S. Raafat A. McCurdy D. Boulanger C. (2012). Genes Affected by Mouse Mammary Tumor Virus (MMTV) Proviral Insertions in Mouse Mammary Tumors Are Deregulated or Mutated in Primary Human Mammary Tumors. Oncotarget 3 (11), 1320–1334. 10.18632/oncotarget.682 23131872 PMC3717796

[B22] Cullen B. R. (2009). Viral and Cellular Messenger RNA Targets of Viral microRNAs. Nature 457, 421–425. 10.1038/nature07757 19158788 PMC3074184

[B23] de Martel C. Ferlay J. Franceschi S. Vignat J. Bray F. Forman D. (2012). Global burden of Cancers Attributable to Infections in 2008: a Review and Synthetic Analysis. Lancet Oncol. 13 (6), 607–615. 10.1016/S1470-2045(12)70137-7 22575588

[B24] Dowran R. Joharinia N. SafaeiBakhtiyarizadeh A. S. Bakhtiyarizadeh S. Alidadi Soleimani A. Alizadeh R. (2019). No Detection of EBV, BKV and JCV in Breast Cancer Tissue Samples in Iran. BMC Res. Notes 12, 171. 10.1186/s13104-019-4178-3 30909983 PMC6434965

[B25] Dyson N. Howley P. M. Münger K. Harlow E. (1989). The Human Papilloma Virus-16 E7 Oncoprotein Is Able to Bind to the Retinoblastoma Gene Product. Science 243 (4893), 934–937. 10.1126/science.2537532 2537532

[B26] El-Sheikh N. Mousa N. O. Tawfeik A. M. Saleh A. M. Elshikh I. Deyab M. (2021). Assessment of Human Papillomavirus Infection and Risk Factors in Egyptian Women with Breast Cancer. Breast Cancer 15, 117822342199627. 10.1177/1178223421996279 PMC7917427 33716506

[B27] Esteller M. Tortola S. Toyota M. Capella G. Peinado M. A. Baylin S. B. (2000). Hypermethylation-Associated Inactivation of p14(ARF) Is Independent of p16(INK4a) Methylation and P53 Mutational Status. Cancer Res. 60 (1), 129–133. 10646864

[B28] Esteller M. (2005). Aberrant DNA Methylation as a Cancer-Inducing Mechanism. Annu. Rev. Pharmacol. Toxicol. 45, 629–656. 10.1146/annurev.pharmtox.45.120403.095832 15822191

[B29] Feldman D. Roniger M. Bar-Sinai A. Braitbard O. Natan C. Love D. C. (2012). The Signal Peptide of Mouse Mammary Tumor Virus-Env: A Phosphoprotein Tumor Modulator. Mol. Cancer Res. 10 (8), 1077–1086. 10.1158/1541-7786.MCR-11-0581 22740636

[B30] Gao A. Kouznetsova V. L. Tsigelny I. F. (2020). Bovine Leukemia Virus Relation to Human Breast Cancer: Meta-Analysis. Microb. Pathogenesis 149, 104417. 10.1016/j.micpath.2020.104417 PMC7384413 32731009

[B31] Ghoncheh M. Mohammadian-Hafshejani A. Salehiniya H. (2015). Incidence and Mortality of Breast Cancer and Their Relationship to Development in Asia. Asian Pac. J. Cancer Prev. 16 (14), 6081–6087. 10.7314/apjcp.2015.16.14.6081 26320499

[B32] Giovanna M. Carlos U. J. María U. A. Gutierrez M. F. (2013). Bovine Leukemia Virus Gene Segment Detected in Human Breast Tissue. Open J. Med. Microbiol. 03 (1), 84–90. 10.4236/ojmm.2013.31013

[B33] Glenn W. K. Heng B. Delprado W. Iacopetta B. Whitaker N. J. Lawson J. S. (2012). Epstein-Barr Virus, Human Papillomavirus and Mouse Mammary Tumour Virus as Multiple Viruses in Breast Cancer. PLoS One 7 (11), e48788. 10.1371/journal.pone.0048788 23183846 PMC3501510

[B34] Gupta I. Jabeen A. Al-Sarraf R. Farghaly H. Vranic S. Sultan A. A. (2020). The Co-Presence of High-Risk Human Papillomaviruses and Epstein-Barr Virus is Linked with Tumor Grade and Stage in Qatari Women with Breast Cancer. Hum. Vaccin. Immunother. 17, 982–989. 10.1080/21645515.2020.1802977 33006291 PMC8018460

[B35] Hameed Y. Usman M. Ahmad M. (2020). Does Mouse Mammary Tumor-Like Virus Cause Human Breast Cancer? Applying Bradford Hill Criteria Postulates. Bull. Natl. Res. Cent. 44, 183. 10.1186/s42269-020-00439-0

[B36] Hedau S. Kumar U. HussainShukla S. S. Shukla S. Pande S. Jain N. (2011). Breast Cancer and Human Papillomavirus Infection: No Evidence of HPV Etiology of Breast Cancer in Indian Women. BMC Cancer 11, 27. 10.1186/1471-2407-11-27 21247504 PMC3036645

[B37] Herrmann K. Niedobitek G. (2002). Lack of Evidence for an Association of Epstein-Barr Virus Infection with Breast Carcinoma. Breast Cancer Res. 5, R13–R17. 10.1186/bcr561 12559053 PMC154138

[B38] Hizi A. Herzig E. (2015). dUTPase: the Frequently Overlooked Enzyme Encoded by many Retroviruses. Retrovirology 12, 70. 10.1186/s12977-015-0198-9 26259899 PMC4531489

[B39] Horakova D. Bouchalova K. Cwiertka K. Stepanek L. Vlckova J. Kollarova H. (2018). Risks and Protective Factors for Triple Negative Breast Cancer with a Focus on Micronutrients and Infections. Biomed. Pap. 162 (2), 83–89. 10.5507/bp.2018.014 29765171

[B40] HPV and Cancer National Cancer Institute (2021). Available at: https://www.cancer.gov/about-cancer/causes-prevention/risk/infectious-agents/hpv-and-cancer (Accessed on April 17, 2021).

[B41] Hu H. Luo M.-L. Desmedt C. Nabavi S. Yadegarynia S. Hong A. (2016). Epstein-Barr Virus Infection of Mammary Epithelial Cells Promotes Malignant Transformation. EBioMedicine 9, 148–160. 10.1016/j.ebiom.2016.05.025 27333046 PMC4972522

[B42] Huo Q. Zhang N. Yang Q. (2012). Epstein-Barr Virus Infection and Sporadic Breast Cancer Risk: A Meta-Analysis. PLoS One 7 (2), e31656. 10.1371/journal.pone.0031656 22363698 PMC3283657

[B43] Islam S. Dasgupta H. Roychowdhury A. Bhattacharya R. Mukherjee N. Roy A. (2017). Study of Association and Molecular Analysis of Human Papillomavirus in Breast Cancer of Indian Patients: Clinical and Prognostic Implication. PLoS One 12 (2), e0172760. 10.1371/journal.pone.0172760 28245287 PMC5330495

[B44] Kadivar M. Monabati A. Joulaee A. Hosseini N. (2011). Epstein-Barr Virus and Breast Cancer: Lack of Evidence for an Association in Iranian Women. Pathol. Oncol. Res. 17 (3), 489–492. 10.1007/s12253-010-9325-z 21207256

[B45] Kang M.-S. Kieff E. (2015). Epstein-Barr Virus Latent Genes. Exp. Mol. Med. 47 (1), e131. 10.1038/emm.2014.84 25613728 PMC4314583

[B46] Katz E. Lareef M. H. Rassa J. C. Grande S. M. King L. B. Russo J. (2005). MMTV *Env* Encodes an ITAM Responsible for Transformation of Mammary Epithelial Cells in Three-Dimensional Culture. J. Exp. Med. 201 (3), 431–439. 10.1084/jem.20041471 15684322 PMC2213037

[B47] Ketterer S. Mitschke J. Ketscher A. Schlimpert M. Reichardt W. Baeuerle N. (2020). Cathepsin D Deficiency in Mammary Epithelium Transiently Stalls Breast Cancer by Interference with mTORC1 Signaling. Nat. Commun. 11 (1), 5133. 10.1038/s41467-020-18935-2 33046706 PMC7552405

[B48] Khalid H. F. Ali A. Fawad N. Rafique S. Ullah I. Rehman G. (2021). MMTV-LIKE Virus and C-Myc Over-expression are Associated with Invasive Breast Cancer. Infect. Genet. Evol. 91, 104827. 10.1016/j.meegid.2021.104827 33794352

[B49] Khalilian M. Hosseini S. M. Madadgar O. (2019). Bovine Leukemia Virus Detected in the Breast Tissue and Blood of Iranian Women. Microb. Pathogenesis 135, 103566. 10.1016/j.micpath.2019.103566 31252065

[B50] Khan N. A. Castillo A. Koriyama C. Kijima Y. Umekita Y. Ohi Y. (2008). Human Papillomavirus Detected in Female Breast Carcinomas in Japan. Br. J. Cancer 99 (3), 408–414. 10.1038/sj.bjc.6604502 18648364 PMC2527789

[B51] Khatami A. Pormohammad A. Farzi R. Saadati H. Mehrabi M. Kiani S. J. (2020). Bovine Leukemia Virus (BLV) and Risk of Breast Cancer: a Systematic Review and Meta-Analysis of Case-Control Studies. Infect. Agents Cancer 15 (1), 1–8. 10.1186/s13027-020-00314-7 PMC7374970 32704306

[B52] Kwong A. Leung C. P. Shin V. Y. Ng E. K. O. (2013). No Evidence of Human Papillomavirus in Patients with Breast Cancer in Hong Kong, Southern China. ISRN Virol. 2013, 1–4. 10.5402/2013/546503

[B53] Labrecque L. G. Barnes D. M. Fentiman I. S. Griffin B. E. (1995). Epstein-Barr Virus in Epithelial Cell Tumors: a Breast Cancer Study. Cancer Res. 55 (1), 39–45. 7805038

[B54] Lawson J. S. (2009). Do Viruses Cause Breast Cancer. Cancer Epidemiol. Methods Mol. Biol. Vol. 471, 421–438. 10.1007/978-1-59745-416-2_21 19109792

[B55] Lawson J. S. Glenn W. K. (2019). Evidence for a Causal Role by Mouse Mammary Tumour-like Virus in Human Breast Cancer. NPJ Breast Cancer 5 (1), 40. 10.1038/s41523-019-0136-4 31728407 PMC6838066

[B56] Lawson J. S. Glenn W. K. (2017). Multiple Oncogenic Viruses Are Present in Human Breast Tissues before Development of Virus Associated Breast Cancer. Infect. Agents Cancer 12, 55. 10.1186/s13027-017-0165-2 PMC5644159 29075317

[B57] Lawson J. S. Glenn W. K. Salyakina D. Clay R. Delprado W. Cheerala B. (2016). Human Papilloma Virus Identification in Breast Cancer Patients with Previous Cervical Neoplasia. Front. Oncol. 5, 298. 10.3389/fonc.2015.00298 26779441 PMC4705232

[B58] Lawson J. S. Glenn W. K. Salyakina D. Delprado W. Clay R. Antonsson A. (2015). Human Papilloma Viruses and Breast Cancer. Front. Oncol. 5, 277. 10.3389/fonc.2015.00277 26734565 PMC4679879

[B59] Lawson J. S. Salmons B. Glenn W. K. (2018). Oncogenic Viruses and Breast Cancer: Mouse Mammary Tumor Virus (MMTV), Bovine Leukemia Virus (BLV), Human Papilloma Virus (HPV), and Epstein-Barr Virus (EBV). Front. Oncol. 8, 1. 10.3389/fonc.2018.00001 29404275 PMC5786831

[B60] Le D. T. Yamashita-Kawanishi N. Okamoto M. Nguyen S. V. Nguyen N. H. Sugiura K. (2020). Detection and Genotyping of Bovine Leukemia Virus (BLV) in Vietnamese Cattle. J. Vet. Med. Sci. 82 (7), 1042–1050. 10.1292/jvms.20-0094 32475959 PMC7399327

[B61] Lehrer S. Rheinstein P. H. (2019). Mouse Mammary Tumor Viral Env Sequences are not Present in the Human Genome but are Present in Breast Tumors and normal Breast Tissues. Virus. Res. 266, 43–47. 10.1016/j.virusres.2019.03.011 30951792 PMC6510484

[B62] Lin J.-H. Tsai C.-H. Chu J.-S. Chen J.-Y. Takada K. Shew J.-Y. (2007). Dysregulation of HER2/HER3 Signaling Axis in Epstein-Barr Virus-Infected Breast Carcinoma Cells. J. Virol. 81 (11), 5705–5713. 10.1128/JVI.00076-07 17376931 PMC1900270

[B63] Liu X. Tang X. Zhang S. Wang Y. Wang X. Zhao C. (20122012). Methylation and Expression of Retinoblastoma and Transforming Growth Factor-Β1 Genes in Epstein-Barr Virus-Associated and -Negative Gastric Carcinomas. Gastroenterol. Res. Pract. 2012, 1–8. 10.1155/2012/906017 PMC3447358 23008701

[B64] Liu Y. Chen J. J. Gao Q. Dalal S. Hong Y. Mansur C. P. (1999). Multiple Functions of Human Papillomavirus Type 16 E6 Contribute to the Immortalization of Mammary Epithelial Cells. J. Virol. 73 (9), 7297–7307. 10.1128/JVI.73.9.7297-7307.1999 10438818 PMC104255

[B65] Loutfy S. A. Abdallah Z. F. Shaalan M. Moneer M. Karam A. Moneer M. M. (2021). Prevalence of MMTV-like Env Sequences and its Association with BRCA1/2 Genes Mutations Among Egyptian Breast Cancer Patients. Cancer Manag. Res. 13 (13), 2835–2848. 10.2147/CMAR.S294584 33814932 PMC8009344

[B66] Magrath I. Bhatia K. (1999). Breast Cancer: A New Epstein-Barr Virus-Associated Disease? J. Natl. Cancer Inst. 91 (16), 1349–1350. 10.1093/jnci/91.16.1349 10451431

[B67] Malekpour Afshar R. Balar N. Mollaei H. R. Arabzadeh S. A. Iranpour M. (2018). Low Prevalence of Human Papilloma Virus in Patients with Breast Cancer, Kerman; Iran. Asian Pac. J. Cancer Prev. 19 (11), 3039–3044. 10.31557/APJCP.2018.19.11.3039 30485938 PMC6318420

[B68] Martinez Cuesta L. Lendez P. A. Nieto Farias M. V. Dolcini G. L. Ceriani M. C. (2018). Can Bovine Leukemia Virus be Related to Human Breast Cancer? A Review of the Evidence. J. Mammary Gland Biol. Neoplasia 23 (3), 101–107. 10.1007/s10911-018-9397-z 29777406

[B69] Mazouni C. Fina F. Romain S. Ouafik L. Bonnier P. Brandone J.-M. (2011). Epstein-Barr Virus as a Marker of Biological Aggressiveness in Breast Cancer. Br. J. Cancer 104 (2), 332–337. 10.1038/sj.bjc.6606048 21179039 PMC3031896

[B70] Mazouni C. Fina F. Romain S. Ouafik L. H. Bonnier P. Martin P.-M. (2015). Outcome of Epstein-Barr Virus-Associated Primary Breast Cancer. Mol. Clin. Oncol. 3 (2), 295–298. 10.3892/mco.2014.459 25798256 PMC4360834

[B71] Morales-Sánchez A. Molina-Muñoz T. Martínez-López J. L. E. Hernández-Sancén P. Mantilla A. Leal Y. A. (2013). No Association Between Epstein-Barr Virus and Mouse Mammary Tumor Virus with Breast Cancer in Mexican Women. Sci. Rep. 3, 2970. 10.1038/srep02970 24131889 PMC3797988

[B72] Motamedifar M. Saki M. Ghaderi A. (2012). Lack of Association of Mouse Mammary Tumor Virus-like Sequences in Iranian Breast Cancer Patients. Med. Princ. Pract. 21 (3), 244–248. 10.1159/000334572 22213781

[B73] Moustafa A.-E. A. (2015). E5 and E6/E7 of High-Risk HPVs Cooperate to Enhance Cancer Progression through EMT Initiation. Cell Adhes. Migration 9 (5), 392–393. 10.1080/19336918.2015.1042197 PMC4955364 26177717

[B74] Münger K. Baldwin A. Edwards K. M. Hayakawa H. Nguyen C. L. Owens M. (2004). Mechanisms of Human Papillomavirus-Induced Oncogenesis. J. Virol. 78 (21), 11451–11460. 10.1128/JVI.78.21.11451-11460.2004 15479788 PMC523272

[B75] Naccarato A. G. Lessi F. Zavaglia K. Scatena C. Al Hamad M. A. Aretini P. (2019). Mouse Mammary Tumor Virus (MMTV) - Like Exogenous Sequences Are Associated with Sporadic but Not Hereditary Human Breast Carcinoma. Aging 11 (17), 7236–7241. 10.18632/aging.102252 31518337 PMC6756874

[B76] Nartey T. Mazzanti C. M. Melana S. Glenn W. K. Bevilacqua G. Holland J. F. (2017). Mouse Mammary Tumor-Like Virus (MMTV) is Present in Human Breast Tissue Before Development of Virally Associated Breast Cancer. Infect. Agents Cancer 12, 1. 10.1186/s13027-016-0113-6 PMC5209856 28053656

[B77] Naushad W. Surriya O. Sadia H. (2017). Prevalence of EBV, HPV and MMTV in Pakistani Breast Cancer Patients: A Possible Etiological Role of Viruses in Breast Cancer. Infect. Genet. Evol. 54, 230–237. 10.1016/j.meegid.2017.07.010 28705719

[B78] Ohba K. Ichiyama K. Yajima M. Gemma N. Nikaido M. Wu Q. (2014). *In Vivo* and *In Vitro* Studies Suggest a Possible Involvement of HPV Infection in the Early Stage of Breast Carcinogenesis via APOBEC3B Induction. PLoS One 9 (5), e97787. 10.1371/journal.pone.0097787 24858917 PMC4032256

[B79] Olaya-Galán N. N. Corredor-Figueroa A. P. Guzmán-garzón T. C. Ríos-hernandez K. S. Salas-cárdenas S. P. Patarroyo M. A. (2017). Bovine Leukaemia Virus DNA in Fresh Milk and Raw Beef for Human Consumption. Epidemiol. Infect. 145 (15), 3125–3130. 10.1017/S0950268817002229 28956522 PMC9148755

[B80] Olson C. Baumgartener L. E. (1976). Pathology of Lymphosarcoma in Sheep Induced with Bovine Leukemia Virus. Cancer Res. 36 (7 pt 1), 2365–2373. 179703

[B81] Pereira Suarez A. L. Lorenzetti M. A. Gonzalez Lucano R. Cohen M. Gass H. Vazquez P. M. (2013). Presence of Human Papilloma Virus in a Series of Breast Carcinoma from Argentina. PLoS One 8 (4), e61613. 10.1371/journal.pone.0061613 23637866 PMC3636204

[B82] Perrigoue J. G. den Boon J. A. Friedl A. Newton M. A. Ahlquist P. Sugden B. (2005). Lack of Association Between EBV and Breast Carcinoma. Cancer Epidemiol. Biomarkers Prev. 14 (4), 809–814. 10.1158/1055-9965.EPI-04-0763 15824148

[B83] Perzova R. Abbott L. BenzLandas P. S. Landas S. Khan S. Glaser J. (2017). Is MMTV Associated with Human Breast Cancer? Maybe, but Probably Not. Virol. J. 14, 196. 10.1186/s12985-017-0862-x 29029634 PMC5640909

[B84] Pfeffer S. Zavolan M. Grässer F. A. Chien M. Russo J. J. Ju J. (2004). Identification of Virus-Encoded microRNAs. Science 304 (5671), 734–736. 10.1126/science.1096781 15118162

[B85] Polyak K. (2007). Breast Cancer: Origins and Evolution. J. Clin. Invest. 117 (11), 3155–3163. 10.1172/JCI33295 17975657 PMC2045618

[B86] Polz-Dacewicz M. Strycharz-Dudziak M. Dworzański J. Stec A. Kocot J. (2016). Salivary and Serum IL-10, TNF-α, TGF-β, VEGF Levels in Oropharyngeal Squamous Cell Carcinoma and Correlation with HPV and EBV Infections. Infect. Agents Cancer 11 1. 10.1186/s13027-016-0093-6 PMC4992298 27547238

[B87] Prabhu S. R. Wilson D. F. (2016). Evidence of Epstein-Barr Virus Association with Head and Neck Cancers: A Review. J. Can. Dent. Assoc. 82, g2. 27548665

[B88] Purdie J. (2019). Can Human Papilloma Virus (HPV) Cause Breast Cancer? Available at: https://www.healthline.com/health/breast-cancer/breast-cancer-and-hpv (Accessed on April 17, 2021).

[B89] Rassi H. Houshmand M. Hashemi M. Majidzadeh A. K. Akbari M. H. H. (2009). Investigation of Mitochondrial Common Deletions and BRCA Mutations for Detection of Familial Breast Cancers in Archival Breast Cancer Materials. Iran J. Cancer Prev. 2 (2), 77–83.

[B90] Richardson A. K. Currie M. J. Robinson B. A. Morrin H. Phung Y. Pearson J. F. (2015). Cytomegalovirus and Epstein-Barr Virus in Breast Cancer. PLoS One 10 (2), e0118989. 10.1371/journal.pone.0118989 25723522 PMC4344231

[B91] Ryan J. L. Jones R. J. Kenney S. C. Rivenbark A. G. Tang W. Knight E. R. (2010). Epstein-Barr Virus-Specific Methylation of Human Genes in Gastric Cancer Cells. Infect. Agents Cancer 5, 27. 10.1186/1750-9378-5-27 PMC3023757 21194482

[B92] Saito S. Kitamura-Muramatsu Y. Komine F. Polat M. Takeshima S.-n. Takei M. (2020). Absence of Bovine Leukemia Virus Proviral DNA in Japanese Human Blood Cell Lines and Human Cancer Cell Lines. Arch. Virol. 165 (1), 207–214. 10.1007/s00705-019-04474-9 31776677

[B93] Salman N. A. Davies G. Majidy F. Shakir F. Akinrinade H. Perumal D. (2017). Association of High Risk Human Papillomavirus and Breast Cancer: A UK Based Study. Sci. Rep. 7, 43591. 10.1038/srep43591 28240743 PMC5378907

[B94] San T. H. Fujisawa M. FushimiYoshimura S. T. Yoshimura T. Ohara T. Soe L. (2017). Low Prevalence of Human Mammary Tumor Virus (HMTV) in Breast Cancer Patients from Myanmar. Infect. Agents Cancer 12, 20. 10.1186/s13027-017-0130-0 PMC5389013 28413435

[B95] Schrauzer G. N. (2006). Interactive Effects of Selenium and Chromium on Mammary Tumor Development and Growth in MMTV-Infected Female Mice and Their Relevance to Human Cancer. Bter 109 (3), 281–292. 10.1385/BTER:109:3:281 16632896

[B96] Schwartz I. Lévy D. (1994). Pathobiology of Bovine Leukemia Virus. Vet. Res. 25 (6), 521–536. 7889034

[B97] Shukla S. Bharti A. C. Mahata S. Hussain S. Kumar R. Hedau S. (2009). Infection of Human Papillomaviruses in Cancers of Different Human Organ Sites. Indian J. Med. Res. 130 (3), 222–233. 19901431

[B98] Sidransky D. Hollstein M. (1996). Clinical Implications of the P53 Gene. Annu. Rev. Med. 47, 285–301. 10.1146/annurev.med.47.1.285 8712782

[B99] Siegel R. L. Miller K. D. Jemal A. (2016). Cancer Statistics, 2016. CA: A Cancer J. Clinicians 66 (1), 7–30. 10.3322/caac.21332 26742998

[B100] Sigaroodi A. Nadji S. A. Naghshvar F. Nategh R. Emami H. Velayati A. A. (2012). Human Papillomavirus Is Associated with Breast Cancer in the North Part of Iran. Scientific World J. 2012, 1–8. 10.1100/2012/837191 PMC3329875 22566779

[B101] Sinha G. (2016). Bovine Leukemia Virus Possibly Linked to Breast Cancer. J. Natl. Cancer Inst. 108 (2), djw020. 10.1093/jnci/djw020 26864929

[B102] Speck P. Longnecker R. (2000). Infection of Breast Epithelial Cells with Epstein-Barr Virus via Cell-to-Cell Contact. J. Natl. Cancer Inst. 92, 1849–1851. 10.1093/jnci/92.22.1849 11078764

[B103] Stewart T. H. M. Sage R. D. Stewart A. F. R. Cameron D. W. (2000). Breast Cancer Incidence Highest in the Range of One Species of House Mouse, Mus Domesticus. Br. J. Cancer 82 (2), 446–451. 10.1054/bjoc.1999.0941 10646903 PMC2363264

[B104] Szakacs J. G. Moscinski L. C. (1991). Sequence Homology of Deoxyribonucleic Acid to Mouse Mammary Tumor Virus Genome in Human Breast Tumors. Ann. Clin. Lab. Sci. 21 (6), 402–412. 1664195

[B105] Szostek S. Zawilinska B. Kopec J. Kosz-Vnenchak M. (2009). Herpesviruses as Possible Cofactors in HPV-16-Related Oncogenesis. Acta Biochim. Pol. 56 (2), 337–342. 10.18388/abp.2009_2466 19499088

[B106] Taneja P. Frazier D. P. Kendig R. D. Maglic D. Sugiyama T. Kai F. (2009). MMTV Mouse Models and the Diagnostic Values of MMTV-like Sequences in Human Breast Cancer. Expert Rev. Mol. Diagn. 9 (5), 423–440. 10.1586/erm.09.31 19580428 PMC2759974

[B107] van Diemen F. R. Kruse E. M. Hooykaas M. J. G. Bruggeling C. E. Schürch A. C. van Ham P. M. (2016). CRISPR/Cas9-Mediated Genome Editing of Herpesviruses Limits Productive and Latent Infections. Plos Pathog. 12 (6), e1005701. 10.1371/journal.ppat.1005701 27362483 PMC4928872

[B108] Vernet-Tomas M. Mena M. Alemany L. Bravo I. De Sanjosé S. Nicolau P. (2015). Human Papillomavirus and Breast Cancer: No Evidence of Association in a Spanish Set of Cases. Anticancer Res. 35 (2), 851–856. 25667466

[B109] Wang J. Quake S. R. (2014). RNA-Guided Endonuclease Provides a Therapeutic Strategy to Cure Latent Herpesviridae Infection. Proc. Natl. Acad. Sci. U.S.A. 111 (36), 13157–13162. 10.1073/pnas.1410785111 25157128 PMC4246930

[B110] Wang Y. Holland J. F. Bleiweiss I. J. Melana S. Liu X. Pelisson I. (1995). Detection of Mammary Tumor Virus *Env* Gene-like Sequences in Human Breast Cancer. Cancer Res. 55 (22), 5173–5179. 7585568

[B111] Wazer D. E. Liu X. L. Chu Q. Gao Q. Band V. (1995). Immortalization of Distinct Human Mammary Epithelial Cell Types by Human Papilloma Virus 16 E6 or E7. Proc. Natl. Acad. Sci. U.S.A. 92 (9), 3687–3691. 10.1073/pnas.92.9.3687 7537374 PMC42026

[B112] Werness B. A. Levine A. J. Howley P. M. (1990). Association of Human Papillomavirus Types 16 and 18 E6 Proteins with P53. Science 248 (4951), 76–79. 10.1126/science.2157286 2157286

[B113] Winer R. L. Lee S. K. Hughes J. P. Adam D. E. Kiviat N. B. Koutsky L. A. (2003). Genital Human Papillomavirus Infection: Incidence and Risk Factors in a Cohort of Female university Students. Am. J. Epidemiol. 157 (3), 218–226. 10.1093/aje/kwf180 12543621

[B114] Yan C. Teng Z. P. Chen Y. X. Shen D. H. Li J. T. Zeng Y. (2016). Viral Etiology Relationship Between Human Papillomavirus and Human Breast Cancer and Target of Gene Therapy. Biomed. Environ. Sci. 29 (5), 331–339. 10.3967/bes2016.043 27353707

[B115] Yarden Y. Sliwkowski M. X. (2001). Untangling the ErbB Signalling Network. Nat. Rev. Mol. Cel Biol. 2 (2), 127–137. 10.38/3505207310.1038/35052073 11252954

[B116] Yuen K.-S. Chan C.-P. Wong N.-H. M. Ho C.-H. Ho T.-H. Lei T. (2015). CRISPR/Cas9-mediated Genome Editing of Epstein-Barr Virus in Human Cells. J. Gen. Virol. 96 (3), 626–636. 10.1099/jgv.0.000012 25502645

[B117] Zekri A.-R. N. Bahnassy A. A. Mohamed W. S. El-Kassem F. A. El-Khalidi S. J. Hafez M. M. (2012). Epstein-Barr Virus and Breast Cancer: Epidemiological and Molecular Study on Egyptian and Iraqi Women. J. Egypt. Natl. Cancer Inst. 24 (3), 123–131. 10.1016/j.jnci.2012.06.001 22929918

[B118] Zhang R. Jiang J. Sun W. Zhang J. Huang K. Gu X. (2016). Lack of Association between Bovine Leukemia Virus and Breast Cancer in Chinese Patients. Breast Cancer Res. 18 (1), 1–2. 10.1186/s13058-016-0763-8 27724949 PMC5057430

[B119] Zhang Y. Fan S. Meng Q. Ma Y. Katiyar P. Schlegel R. (2005). BRCA1 Interaction with Human Papillomavirus Oncoproteins. J. Biol. Chem. 280 (39), 33165–33177. 10.1074/jbc.M505124200 15983032

